# Sharp & Dohme

**Published:** 1881-10-20

**Authors:** 


### EDITORIALS AND MISCELLANEOUS.

*Sharpe & Dohme.*—This excellent and reliable Baltimore house has an advertisement in this Journal. Be sure and examine it.

